# GenePioneer: a comprehensive Python package for identification of essential genes and modules in cancer

**DOI:** 10.1093/bioadv/vbaf094

**Published:** 2025-04-29

**Authors:** Amirhossein Haerianardakani, Golnaz Taheri

**Affiliations:** Department of Computer and Systems Sciences, Stockholm University, Stockholm 16455, Sweden; School of Electrical Engineering and Computer Science, SciLifeLab, KTH Royal Institute of Technology, Stockholm 10044, Sweden

## Abstract

**Summary:**

We propose a network-based unsupervised learning model to identify essential cancer genes and modules for 12 different cancer types, supported by a Python package for practical application. The model constructs a gene network from frequently mutated genes and biological processes, ranks genes using topological features, and detects critical modules. Evaluation across cancer types confirms its effectiveness in prioritizing cancer-related genes and uncovering relevant modules. The Python package allows users to input gene lists, retrieve rankings, and identify associated modules. This work provides a robust method for gene prioritization and module detection, along with a user-friendly package to support research and clinical decision-making in cancer genomics.

**Availability and implementation:**

GenePioneer is released as an open-source software under the MIT license. The source code is available on GitHub at https://github.com/Golnazthr/ModuleDetection.

## 1 Introduction

Cancer arises from alterations in genes, nucleotides, and cellular structures. Somatic cells, mutating an order of magnitude faster than germline cells, are more susceptible to cancer (Lynch 2010). Mutations can modify protein function and alter diverse cellular processes, leading to significant intra- and inter-tumor heterogeneity in biochemistry and histology (Dagogo-Jack and Shaw 2018). This heterogeneity complicates cancer treatment and the identification of causative events. The recent focus on mutations underscores the importance of gene-specific analysis to identify cancer driver genes (McLendon *et al.* 2008). Identifying recurrently mutated genes can aid in predicting cancer progression. However, many cancer-driver genes remain elusive, with many mutations undetected by current methods. Recent studies have revealed novel genes and cancer gene classes. A comprehensive catalog of mutations in different frequency range is crucial for identifying dysregulated pathways and potential therapeutic targets (Lawrence *et al.* 2014). However, cancer genes often disrupt a limited number of pathways, particularly those involved in survival, cell division, differentiation, and genome maintenance. Therefore, assessing the pathway-level importance of genes, even those with intermediate or low mutation frequencies, is essential. Despite extensive research into critical genes and cancer-related modules for specific cancer types, a significant knowledge gap persists: most studies focus on isolated cancer types. This specialization limits the application of essential gene identifier models across different cancers (Shimada *et al.* 2019). In cancer research, studying gene networks instead of individual genes offers a more comprehensive understanding of cancer development and progression (Schulte *et al.* 2021). Genes operate within complex molecular pathways, and analyzing their interactions helps identify key regulators of critical cellular functions. This approach helps uncover disease subtypes, discover biomarkers, and highlight dysregulated pathways that could serve as potential therapeutic targets. Despite the growing availability of advanced scientific tools, a comprehensive toolkit for identifying essential genes and cancer-related modules remains unavailable. Such a tool should be rooted in scientific research while remaining accessible to researchers with varying levels of expertise. This underscores the need for an integrated solution that connects advanced bioinformatics techniques with practical applications in cancer genetics. To fill this gap, we introduce GenePioneer, a Python package designed to address these challenges.

## 2 Methods

This work proposes a toolkit based on a two-step technique to identify driver genes and modules in cancers. The results for 12 cancer types have been tested and presented, including adrenal, bladder, brain, cervix, colon, corpus uteri, kidney, liver and thyroid. Moreover, this toolkit can incorporate data from other cancer types and identify driver genes and modules for them. In the first step, a network is constructed using biological process terms and a set of mutated genes for each cancer. The Laplacian Score algorithm (LS) is then used to score genes within this network, and a subset of informative genes with high scores is selected for each cancer (Taheri and Habibi 2022). In the second step, Monte Carlo Gene Cluster (MG) is used as a heuristic algorithm to identify the clusters with the highest scores as candidate modules (Habibi and Taheri 2022). Significant clusters with high *P*-values, based on cancer-related pathways, are then identified as a set of cancer-related modules.

### 2.1 Datasets

For the first step, we collected a list of the most frequent mutations for 12 cancer types from the Cancer Genome Atlas Program (TCGA) dataset (McLendon *et al.* 2008). We further enriched the TCGA data with additional biological information from the Gene Ontology (GO) dataset (Gene Ontology Consortium 2019) from UniProt, a public dataset containing biological processes and their associated genes (UniProt Consortium 2019). By incorporating this enriched dataset, we ensure that the proposed Python package is grounded in robust biological data and can uncover meaningful insights into cancer genomics. For evaluating the genes obtained from the first step, we utilized four benchmark datasets CGC (Habibi and Taheri 2022), Rule (Habibi and Taheri 2022), HCD (Habibi and Taheri 2022), and CTAT (Habibi and Taheri 2022), respectively. These benchmarks serve as a reliable reference set of known cancer-related genes.

### 2.2 Network construction

**Figure 1.**
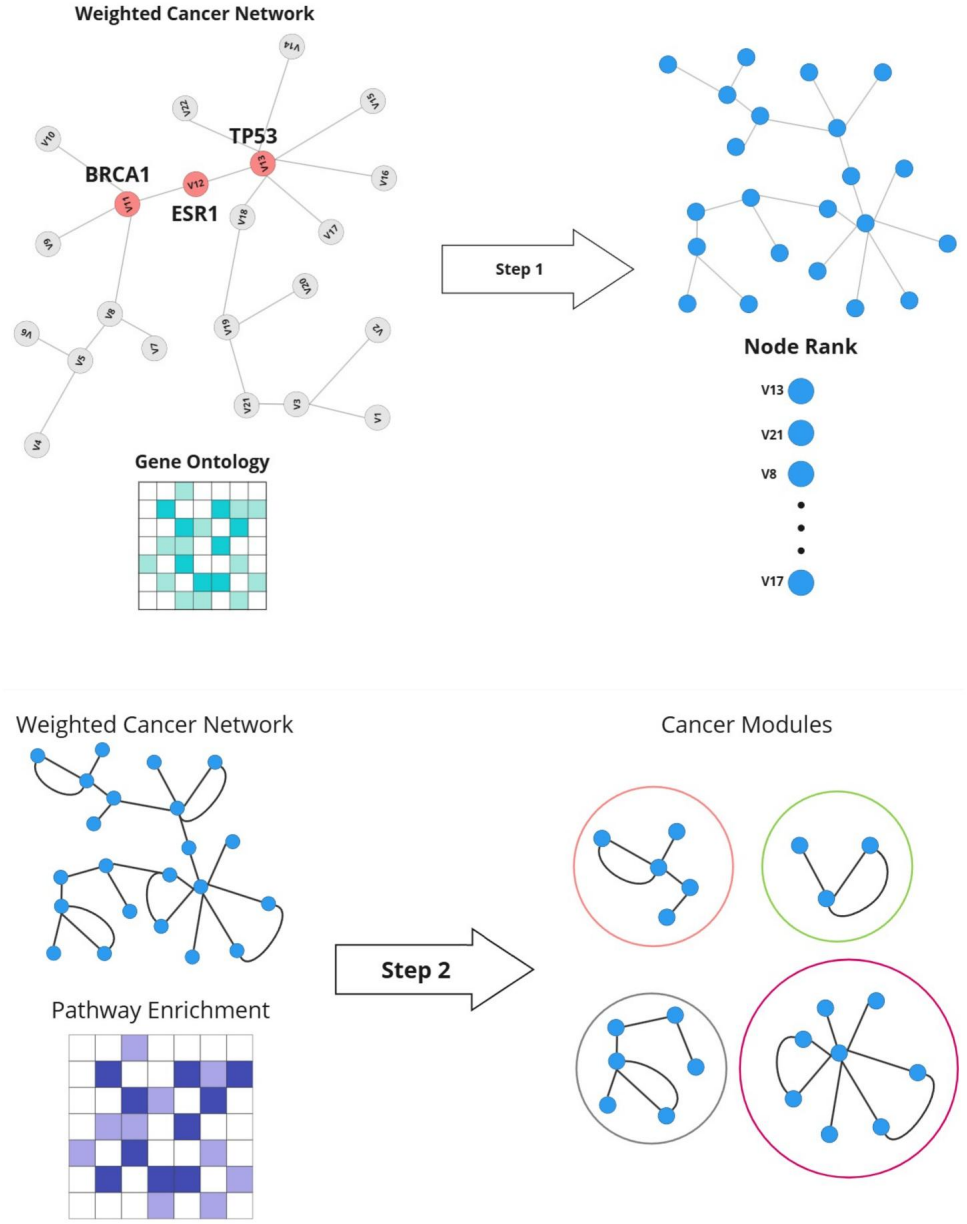
The workflow consists of (i) detecting and ranking important genes and (ii) identifying informative modules. In (i), a weighted gene network is constructed and genes are ranked based on key topological features. In (ii), the MG algorithm is applied to identify densely connected modules, starting from high-ranking seed genes and expanding iteratively.

To construct a biologically meaningful network for gene ranking and module detection, we built an undirected weighted graph G=(V,E,W). The selection of genes (*V*) was based on the idea that frequently mutated genes in a given cancer type are likely to play key roles in its progression. We started with the top 200 most frequently mutated genes ($V_{1}$) for each cancer type. However, since cancer is driven by complex molecular interactions rather than individual genes acting in isolation, we expanded this set by including genes that participate in the same biological processes ($V_{2}$). This ensures that the network not only reflects mutational patterns but also captures functionally relevant relationships between genes. Edges (*E*) were established between genes that share at least one biological process, creating connections based on functional similarity rather than physical interactions alone. Edge weights were determined by the number of shared processes, allowing the network to highlight genes that work closely together in key pathways. To assess the role of each gene within this structure, we calculated the weight, betweenness, centrality of the eigenvector, and entropy of five topological characteristics, each of which captures different aspects of the influence of genes within the network. These features were then ranked using the LS method, which prioritizes genes that maintain strong local connectivity and are more likely to be functionally important. Once gene ranking was established, we applied the MG algorithm to detect functional modules. This algorithm begins with a highly ranked gene as a seed and iteratively expands the module by adding genes that enhance its connectivity. The process continues until no further improvement is possible or a predefined size threshold is met. By identifying these tightly connected modules, the approach helps uncover gene groups that may collectively contribute to cancer progression. The entire workflow, including gene ranking and module detection, is summarized in Fig. 1.

## 3 Results

In this study, we presented two key findings. First, we evaluated the performance of our selected high-score genes by comparing them to four benchmark datasets. Second, we assessed the proposed functional modules using pathway enrichment analysis and calculated a *P*-value to determine their significance in cancer-related pathways.

### 3.1 Evaluation of high-score selected genes

**Figure 2.**
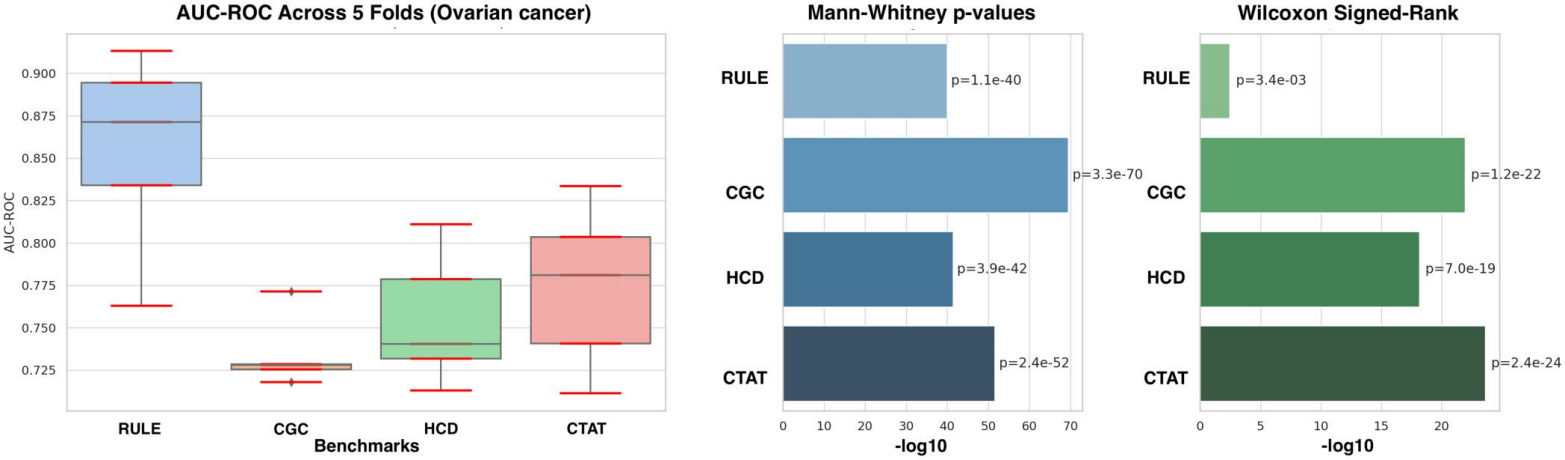
Visualization for AUC-ROC, Mann–Whitney *U* test, and Wilcoxon signed-rank test for each benchmark.

For the first evaluation part, we used area under the receiver operating characteristic (AUC-ROC) and the Mann–Whitney *U* test (Mann and Whitney 1947, Fawcett 2006). To calculate the AUC-ROC, we identified the top *N* genes from the ranked list, where *N* equals the size of the benchmark gene set. A binary classification was assigned to each gene: 1 for genes within the top *N* (predicted as significant with high score) and 0 otherwise. The benchmark gene set served as the ground truth, with genes labeled 1 for significance and 0 for non-significance. Table 1 presents the evaluation results for ovarian cancer as an example, alongside other benchmark methods. As shown in Table 1, a 5-fold cross-validation was performed, and the AUC-ROC for each fold was computed to assess the model’s ability to distinguish between important and non-important genes in a binary classification setting. To further evaluate gene rankings, we applied the Mann–Whitney *U* test, which compares the ranks of benchmark genes to non-benchmark genes within the high-score-ranked gene list. Benchmark gene ranks indicate their position in the list, while other ranks represent non-benchmark genes. A low *P*-value suggests that benchmark genes are consistently ranked higher, demonstrating that the model effectively prioritizes them. Additionally, we computed the Wilcoxon signed-rank test to assess the statistical significance of the model’s gene rankings compared to random rankings. Figure 2 also presents a visualization to facilitate the comparison of evaluation results across different benchmarks. It is important to note that the AUC-ROC values are not extremely high because the existing benchmarks identify broad cancer-related genes rather than cancer-type-specific ones. These benchmarks aim to be as general as possible, selecting large sets of genes that are broadly relevant to cancer. In contrast, our method is designed to identify genes specific to each cancer type, making direct comparisons with these general benchmarks less precise. However, since these are the only relevant benchmarks available, we include them for reference. The evaluation results for other cancer types are provided in Supplementary Table S3.

**Table 1.** The values of our evaluation measure on different benchmarks for ovarian cancer.

Bench	AUC-ROC	Mann-Whit	Wilcoxon
RULE	0.871, 0.834, 0.913, 0.763, 0.894	1.07 × 10${}^{-40}$	3.43 × 10${}^{-3}$
CGC	0.729, 0.718, 0.725, 0.728, 0.771	3.34 × 10${}^{-70}$	1.16 × 10${}^{-23}$
HCD	0.779, 0.713, 0.732, 0.740, 0.811	3.92 × 10${}^{-42}$	7.03 × 10${}^{-19}$
CTAT	0.781, 0.803, 0.741, 0.711, 0.833	2.43 × 10${}^{-52}$	2.44 × 10${}^{-24}$

### 3.2 Evaluation of modules

Now, a set of genes with corresponding rankings is available to identify key genes for each cancer type. As mentioned earlier, genes in cancer typically function within complex molecular patterns rather than acting individually. Therefore, using the high-ranked genes from the previous section, this step focuses on identifying informative modules that contribute to cancer progression. To ensure biological relevance and minimize redundancy, identified modules were refined through a series of steps. Modules were constrained to include 4–10 genes, striking a balance between specificity and comprehensiveness (Taheri *et al.* 2023). Overlapping modules were compared based on their composite scores, retaining only the highest-scoring ones. Finally, quality assessments removed redundant subsets of larger modules, ensuring distinctiveness and clarity.

**Figure 3.**
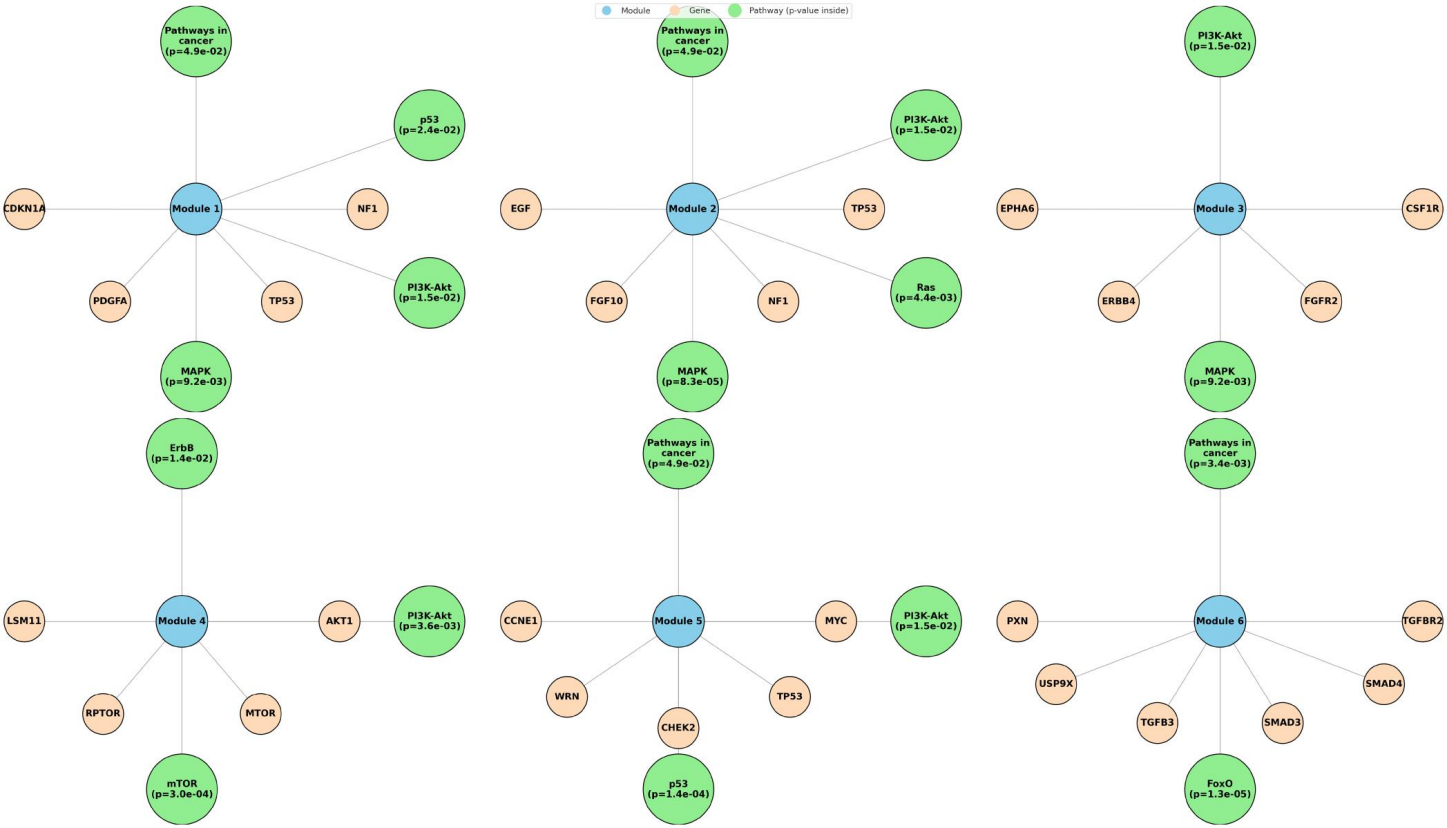
A set of important modules for ovarian cancer was extracted through the proposed method.

The evaluation process assessed each module for biological significance. Genes from each module were analyzed through pathway enrichment profiling using the KEGG database (Kanehisa *et al.* 2016), identifying significant associations based on *P*-values ≤0.05. For this purpose, we used Gene Set Enrichment Analysis (GSEA) method to assess the significance of predefined gene sets based on well-known cancer-relatedpathways (Taheri and Habibi 2024). We calculated the *P*-value as $P–\mathrm{value}=\frac{\left(\begin{array}{c} k\\ K \end{array}\right)\left(\begin{array}{c} n-k\\ N-K \end{array}\right)}{\left(\begin{array}{c} n\\ N \end{array}\right)}.$ Where, *N*, shows the total number of genes in the dataset for each cancer. *K* is the number of genes in a cancer-related pathway $\left(P_{i}\right)$, *n* is the number of driver genes in the detected module (m) and *k* is the number of genes shared between the driver module (m) and the pathway $\left(P_{i}\right)$, respectively. A predefined list of 11 signaling pathways was selected based on their roles in regulating key cellular processes such as survival, proliferation, migration, differentiation, and apoptosis, all of which contribute to cancer progression. These pathways include the FoxO pathway, which regulates cell death, cell cycle, and metabolism; the Wnt pathway, crucial for cancer development and cell migration; and the MAPK pathway, involved in cell survival and proliferation. The p53 pathway controls the cell cycle and apoptosis, often altered in cancers. Estrogen pathway Regulates tissue growth and development, and is linked to some cancers. Pathways in cancer, encompasses various signaling pathways involved in cancer progression. Pathways like Ras, ErbB, and PI3K-Akt govern key cellular functions and are frequently mutated in cancers. The VEGF pathway supports tumor growth through angiogenesis, and the mTOR pathway influences cell growth and metabolism. Together, these pathways offer a comprehensive view of cancer biology. In our evaluation in this part, a module was marked as biologically significant if it showed enrichment in at least two relevant pathways, ensuring statistical and contextual relevance in cancer analysis. Figure 3 displays a part of modulated genes for ovarian cancer. As shown in Fig. 3, the second module includes EGF, FGF10, NF1, and TP53, which are interconnected in the regulation of critical cancer-related pathways such as MAPK, Ras, and PI3K-Akt. Dysregulation of these genes, particularly in ovarian cancer, contributes to tumor progression by enhancing cell proliferation, survival, and migration. The complete module set for ovarian cancer and the results of this part for other 12 cancer types are reported in Supplementary Tables S5–S16.

## 4 Implementation

The GenePioneer workflow integrates these five tools into a seamless pipeline. DataLoader first processes raw data, which NetworkBuilder then transforms into a structured gene network with weighted edges. NetworkAnalysis ranks genes and detects significant modules using network topology and clustering methods. Researchers can explore specific genes through GeneAnalysis, while Evaluation ensures reliability by benchmarking against known datasets. Together, these components enable a comprehensive, efficient analysis of essential cancer genes and modules. Detailed information on each tool and its specific role in the analysis pipeline is provided below.

- DataLoader: Handles the extraction and preprocessing of datasets required for network construction and analysis. This tool ensures seamless loading and mapping of genes, cases, and processes, creating a foundation for downstream analyses.
- NetworkBuilder: Constructs a weighted, undirected gene network by leveraging shared biological processes as edge weights. It enhances the network with topological features, including node weights, centrality metrics, and entropy.
- NetworkAnalysis: Conducts in-depth network analysis to rank genes and detect significant modules. It utilizes LS for feature selection and applies a heuristic module-detection algorithm to identify biologically relevant clusters, ensuring high-quality module detection.
- GeneAnalysis: Enables the detailed exploration of individual genes within the network. With the help of this tool, users can query genes to obtain their rank, associated modules, and topological features.
- Evaluation: Validates the performance of the network and modules against benchmark datasets. It uses metrics such as AUC-ROC, and Mann–Whitney *U* test, alongside pathway enrichment analysis, to assess the biological relevance and accuracy of the identified genes and modules.

The GenePioneer package is a groundbreaking resource for cancer genomics, providing tools to rank essential genes and identify cancer-related modules across various cancer types using unsupervised network-based methods. Accessible via GitHub and PyPi, the toolkit is designed with user-friendliness in mind, enabling researchers and clinicians to gain actionable insights without requiring extensive computational expertise.

## 5 Conclusion

The GenePioneer toolkit was developed as a fast and straightforward way to integrate gene ranking and module detection into a practical, Python-based tool for cancer researchers. It requires minimal input, delivers clear output, and can be run within a Python environment, making it highly user-friendly and accessible to non-expert programmers while supporting large-scale dataset analysis. By evaluating gene importance and identifying gene interactions within cancer networks, GenePioneer provides critical insights into the genetic drivers of cancer. Key features include ranking genes by their network significance and identifying the modules they belong to, which helps explore cancer-related pathways and aids in developing precise therapies. Available on GitHub and PyPi, GenePioneer’s user-centric design ensures that researchers of all skill levels can make use of its capabilities. By combining comprehensive data integration, advanced network-based analysis, and statistical rigor, GenePioneer stands as a versatile and impactful resource for cancer research across multiple cancer types.

## Supplementary Material

vbaf094_Supplementary_Data

## Data Availability

The GenePioneer source code is freely available at https://github.com/Golnazthr/ModuleDetection.
