# Supplementary material for: GenePioneer: a comprehensive Python package for identification of essential genes and modules in cancer
Source: Bioinform Adv. 2025 Apr 29;5(1):vbaf094. doi: 10.1093/bioadv/vbaf094 (PMC12098931; doi:10.1093/bioadv/vbaf094)
Supplement: vbaf094_Supplementary_Data [file vbaf094_supplementary_data.pdf]

Genepioneer supplementary data

Table 1 shows the detailed information about the constructed network for various cancer types.

Cancer Type	Number of Genes	Number of Edges	Average Degree	Density
Ovary	9915	747697	150.82	0.015
Adrenal	10087	748954	148.5	0.014
Bladder	9818	738006	150.34	0.015
Brain	9814	743361	151.49	0.015
Cervix	10244	789463	154.13	0.015
Colon	10169	779012	153.21	0.015
Corpus Uteri	9811	728780	148.56	0.015
Kidney	10038	745836	148.6	0.014
Liver	9884	727582	147.22	0.014
Prostate	9739	748707	153.75	0.015
Skin	9957	766284	153.92	0.015
Thyroid	10157	771949	152.0	0.015

Table 1 - An overview of the network for each cancer type, including detailed information about each cancer.

Table 2 shows the top 5 genes with the highest LS score for each cancer type.

Gene	Weight	Closeness	Betweenness	Eigenvector	Effect on entropy	LS score	Cancer type
ABL1	18722	0.546	0.012	0.073	0.002	60.792	Colon
TP53	18908	0.538	0.011	0.065	0.002	58.495	
TGFB1	19340	0.533	0.005	0.069	0.003	52.138	
NF1	18648	0.535	0.006	0.065	0.003	50.954	
CTNNB1	16998	0.535	0.007	0.064	0.002	48.199	
TP53	19280	0.539	0.011	0.067	0.003	69.429	Kidney
ABL1	18482	0.546	0.012	0.075	0.003	69.176	
MTOR	14664	0.544	0.011	0.065	0.002	53.801	
NF1	16362	0.535	0.007	0.066	0.002	52.760	
TNF	14226	0.533	0.005	0.070	0.002	44.777	
ABL1	19308	0.550	0.012	0.073	0.003	63.377	Prostate
AKT1	18342	0.552	0.013	0.072	0.002	61.312	
TP53	19418	0.543	0.011	0.064	0.003	60.193	
MTOR	14668	0.547	0.011	0.062	0.001	47.292	
TGFB1	17688	0.536	0.005	0.069	0.003	47.115	
TP53	20634	0.538	0.010	0.064	0.003	64.847	Thyroid
ABL1	17896	0.548	0.012	0.072	0.002	61.103	
AKT1	17100	0.548	0.013	0.072	0.002	59.428	
SRC	18658	0.539	0.008	0.069	0.003	57.634	
TNF	18400	0.542	0.006	0.075	0.003	53.577	
TP53	18628	0.541	0.011	0.067	0.003	65.311	Liver
NF1	18376	0.538	0.007	0.066	0.003	57.275	
MTOR	12674	0.544	0.012	0.065	0.001	47.240	
CTNNB1	14458	0.536	0.007	0.065	0.002	46.956	
TGFB1	15098	0.533	0.006	0.071	0.002	46.852	
TP53	21468	0.541	0.011	0.066	0.003	70.229	Bladder
ABL1	18918	0.549	0.012	0.074	0.002	65.149	
NF1	18992	0.538	0.007	0.066	0.003	55.313	

MTOR	15382	0.545	0.011	0.064	0.001	51.258	
CTNNB1	15228	0.537	0.007	0.064	0.002	46.183	
TP53	18120	0.540	0.011	0.065	0.002	61.080	Brain
ABL1	15000	0.542	0.009	0.068	0.002	49.662	
MTOR	14000	0.546	0.011	0.063	0.001	49.243	
NF1	16306	0.537	0.007	0.065	0.002	47.986	
GATA3	15352	0.530	0.006	0.061	0.002	43.809	
ABL1	17622	0.546	0.013	0.074	0.003	65.868	Adrenal
TP53	17922	0.539	0.011	0.066	0.003	63.109	
CTNNB1	15516	0.535	0.007	0.065	0.002	50.104	
NF1	15936	0.535	0.007	0.066	0.002	50.102	
TGFB1	15632	0.532	0.005	0.071	0.002	48.724	
TP53	20224	0.541	0.010	0.064	0.003	68.206	Cervix
ABL1	17172	0.546	0.011	0.072	0.002	60.262	
NF1	17530	0.537	0.006	0.063	0.002	52.547	
MTOR	15534	0.542	0.010	0.062	0.002	52.311	
TGFB1	16196	0.533	0.005	0.066	0.002	48.205	
TP53	19424	0.541	0.011	0.066	0.003	70.153	Ovary
MTOR	14250	0.544	0.011	0.064	0.001	52.650	
NF1	16348	0.538	0.007	0.066	0.002	51.916	
GATA3	15316	0.531	0.006	0.062	0.002	47.246	
TNF	14198	0.542	0.006	0.073	0.002	45.828	
ABL1	20152	0.548	0.012	0.073	0.003	65.987	Skin
TP53	18490	0.541	0.011	0.064	0.002	58.623	
SRC	16638	0.535	0.007	0.066	0.002	49.203	
MTOR	14116	0.544	0.011	0.062	0.001	46.399	
NF1	16890	0.537	0.006	0.064	0.002	46.366	
TP53	17522	0.541	0.012	0.067	0.003	65.291	Corpus uteri
NF1	16760	0.537	0.007	0.067	0.003	54.945	
MTOR	12810	0.545	0.012	0.065	0.001	49.848	

NOTCH1	14838	0.531	0.007	0.062	0.002	49.400
CTNNB1	13844	0.537	0.008	0.066	0.002	47.760

Table 2 - top 5 genes with the highest LS scores for each cancer type, highlighting their potential significance in cancer progression.

Table 3 shows the results of the evaluation for **AUC-ROC** and **Mann-Whitney U Test for each cancer type and each benchmark.**

Cancer Type	Benchmark	AUC-ROC	Mann-Whitney U Test P-value
**Colon**	RULE	0.632	4.745×10^{-41}
	CGC	0.618	1.246×10^{-75}
	HCD	0.594	3.332×10^{-42}
	CTAT	0.617	2.628×10^{-55}
**Kidney**	RULE	0.614	7.062×10^{-39}
	CGC	0.621	4.948×10^{-70}
	HCD	0.594	1.637×10^{-41}
	CTAT	0.623	3.278×10^{-54}
**Prostate**	RULE	0.617	5.499×10^{-37}
	CGC	0.616	4.503×10^{-66}
	HCD	0.585	1.141×10^{-38}
	CTAT	0.613	4.049×10^{-51}
**Thyroid**	RULE	0.621	2.988×10^{-58}
	CGC	0.612	6.056×10^{-64}
	HCD	0.592	1.671×10^{-41}
	CTAT	0.615	2.239×10^{-54}
**Liver**	RULE	0.633	3.018×10^{-42}
	CGC	0.62	4.312×10^{-74}
	HCD	0.592	7.640×10^{-42}
	CTAT	0.63	1.721×10^{-54}
**Bladder**	RULE	0.624	2.325×10^{-40}
	CGC	0.624	4.789×10^{-73}

	HCD	0.595	2.560×10^{-41}
	CTAT	0.624	1.795×10^{-56}
**Brain**	RULE	0.627	9.910×10^{-40}
	CGC	0.62	8.332×10^{-70}
	HCD	0.594	9.339×10^{-40}
	CTAT	0.622	4.199×10^{-53}
**Adrenal**	RULE	0.611	1.780×10^{-40}
	CGC	0.618	7.692×10^{-72}
	HCD	0.589	6.620×10^{-40}
	CTAT	0.624	1.411×10^{-55}
**Cervix**	RULE	0.628	2.772×10^{-40}
	CGC	0.619	4.944×10^{-72}
	HCD	0.6	6.696×10^{-45}
	CTAT	0.623	2.836×10^{-56}
**Ovary**	RULE	0.611	1.076×10^{-40}
	CGC	0.62	3.343×10^{-70}
	HCD	0.595	3.924×10^{-42}
	CTAT	0.622	2.433×10^{-52}
**Skin**	RULE	0.606	6.636×10^{-39}
	CGC	0.611	9.796×10^{-67}
	HCD	0.587	1.702×10^{-39}
	CTAT	0.615	4.234×10^{-52}
**Corpus**	RULE	0.629	4.197×10^{-41}
	CGC	0.618	9.278×10^{-71}
	HCD	0.599	1.604×10^{-38}
	CTAT	0.629	5.299×10^{-52}

Table 3 - The values of our evaluation measure on different benchmarks for each cancer type.

Table 4 - the number of founded modulesTable 4 shows the numbers of successfully identified gene modules across various cancer types.

Cancer Type	Number of modules
**Brain**	460
**Colon**	567
**Skin**	446
**Adrenal**	338
**Bladder**	530
**Cervix**	497
**Corpus Uteri**	593
**Kidney**	388
**Liver**	482
**Ovary**	459
**Prostate**	516
**Thyroid**	507

Table 4 - the number of founded modules in each cancer type.

Table 5-16 show the evaluation results for the Bladder, Ovary, Cevix, Adrenal, Kidney, Corpus uteri, Prostate, Brain, Liver, Thyroid, Colon, and Skin, cancer types respectively. Each table showing the number of pathways each cancer module participates in, along with the corresponding p-value for the GSEA method.

Module Genes	Significant Pathways	P-value	Number of Pathways
AKT1, SRC, NF1, CTNNB1, ABL1, TP53	ErbB	8.99×10^{-4}	5
	Pathways in cancer	9.78×10^{-3}	
	Ras	1.93×10^{-2}	
	VEGF	3.43×10^{-2}	
	MAPK	4.00×10^{-2}	
ROR1, FLT1, NTRK3, NTRK2, ERBB4	MAPK	2.05×10^{-2}	2
	PI3K-Akt	3.37×10^{-2}	
CDK2, CDK1, CDKN2A, CDKN1A	p53	2.86×10^{-7}	2
	Pathways in cancer	4.88×10^{-2}	
NOA1, PIK3CA, VEGFA, AKT1, IGF1	Ras	3.08×10^{-5}	8
	VEGF	6.30×10^{-5}	
	PI3K-Akt	1.64×10^{-4}	
	FoxO	7.64×10^{-4}	
	Pathways in cancer	7.91×10^{-4}	
	mTOR	1.26×10^{-3}	
	MAPK	9.25×10^{-3}	
	ErbB	3.08×10^{-2}	
MYDGF, UNC5B, VEGFA, ANGPT1, KDR	Ras	4.41×10^{-3}	4
	MAPK	9.25×10^{-3}	
	VEGF	1.51×10^{-2}	
	PI3K-Akt	1.52×10^{-2}	
RERG, KSR1, HRAS, CDKN1A	ErbB	1.42×10^{-2}	2
	FoxO	3.47×10^{-2}	

PREX2, PIK3CA, EDN1, PTEN	Pathways in cancer	1.18×10^{-2}	3
	FoxO	3.47×10^{-2}	
	mTOR	4.86×10^{-2}	
CACNB4, MAP2K1, MAPK1, MAPK3	VEGF	1.58×10^{-5}	9
	ErbB	4.63×10^{-5}	
	FoxO	1.78×10^{-4}	
	Estrogen	2.00×10^{-4}	
	mTOR	2.96×10^{-4}	
	Ras	1.03×10^{-3}	
	MAPK	2.19×10^{-3}	
	PI3K-Akt	3.62×10^{-3}	
	Pathways in cancer	1.18×10^{-2}	
CCRL2, MAPK1, PBX1, MAPK3, ABL1	ErbB	2.00×10^{-4}	4
	Ras	4.41×10^{-3}	
	VEGF	1.51×10^{-2}	
	Pathways in cancer	4.88×10^{-2}	
OR51E2, MST1R, KIT, FGFR3, FLT3, EPHA1	Ras	1.93×10^{-2}	2
	MAPK	4.00×10^{-2}	
PTPRG, EPHA4, BCL10, TEK, RET, IGF1R, INSR	MAPK	1.08×10^{-3}	3
	PI3K-Akt	2.09×10^{-3}	
	Ras	1.93×10^{-2}	
SP1, IRS1, PDPK1, PPARG, AKT1	FoxO	1.72×10^{-3}	3
	mTOR	2.85×10^{-3}	
	PI3K-Akt	3.37×10^{-2}	
TIMM50, BAD, CASP2, BAK1, BCL2L1, BCL2	Pathways in cancer	3.43×10^{-3}	3
	PI3K-Akt	3.37×10^{-2}	
	p53	3.61×10^{-2}	

PMF1, SMC5, BRCA1, BRCA2, CDKN1A, CDKN1B	PI3K-Akt<br>ErbB<br>Pathways in cancer	1.52×10^{-2}<br>3.08×10^{-2}<br>4.88×10^{-2}	3
FAS, MAGED1, FLT1, FGFR1, ERBB2, IGF1R	MAPK<br>PI3K-Akt<br>Pathways in cancer<br>Ras	1.55×10^{-5}<br>2.09×10^{-3}<br>9.78×10^{-3}<br>1.93×10^{-2}	4
SIX6OS1, SYK, PTPN6, FGFR3, KIT, TEK	PI3K-Akt<br>Ras<br>MAPK	7.23×10^{-3}<br>9.87×10^{-3}<br>2.05×10^{-2}	3

Table 5 - Pathway Associations and GSEA p-values for cancer modules in Bladder cancer.

**Module Genes**	**Significant Pathways**	**P-value**	**Number of Pathways**
CDKN1A, PDGFA, TP53, NF1	MAPK<br>PI3K-Akt<br>p53<br>Pathways in cancer	9.25×10^{-3}<br>1.52×10^{-2}<br>2.39×10^{-2}<br>4.88×10^{-2}	4
EGF, FGF10, NF1, TP53	MAPK<br>Ras<br>PI3K-Akt<br>Pathways in cancer	8.34×10^{-5}<br>4.41×10^{-3}<br>1.52×10^{-2}<br>4.88×10^{-2}	4
EPHA6, ERBB4, FGFR2, CSF1R	MAPK<br>PI3K-Akt	9.25×10^{-3}<br>1.52×10^{-2}	2
LSM11, RPTOR, MTOR, AKT1	mTOR<br>PI3K-Akt<br>ErbB	2.96×10^{-4}<br>3.62×10^{-3}<br>1.42×10^{-2}	3
CCNE1, WRN, CHEK2, TP53, MYC	p53<br>PI3K-Akt	1.36×10^{-4}<br>1.52×10^{-2}	3

	Pathways in cancer	4.88×10^{-2}	
PXN, USP9X, TGFB3, SMAD3, SMAD4, TGFBR2	FoxO<br>Pathways in cancer	1.31×10^{-5}<br>3.43×10^{-3}	2
RASGRP3, CDKN2A, HRAS, CDKN1A	Pathways in cancer<br>p53<br>ErbB	7.91×10^{-4}<br>2.39×10^{-2}<br>3.08×10^{-2}	3
CEMP1, SPHK1, CRK, AKT1, IGF1	MAPK<br>VEGF<br>ErbB<br>Pathways in cancer	9.25×10^{-3}<br>1.51×10^{-2}<br>3.08×10^{-2}<br>4.88×10^{-2}	4
IFIT2, AXL, MET, DDR1, ERBB4, EPHA1, PDGFRA	MAPK<br>PI3K-Akt	2.05×10^{-2}<br>3.37×10^{-2}	2
IFNA16, ITGA4, FLT3, ITGB1, KIT	PI3K-Akt<br>Pathways in cancer	6.20×10^{-6}<br>3.43×10^{-3}	2
GPR143, PLCE1, CRKL, PARK7, JUN, TP53	MAPK<br>ErbB	2.05×10^{-2}<br>4.65×10^{-2}	2
PNKP, MAPKAPK5, RALB, IGF1, JUN, HRAS, LIG4, CDKN1A, FGF2	Pathways in cancer<br>MAPK<br>Ras<br>ErbB<br>FoxO<br>PI3K-Akt	2.16×10^{-4}<br>3.29×10^{-4}<br>3.50×10^{-3}<br>4.07×10^{-4}<br>1.52×10^{-2}<br>1.75×10^{-2}	6

Table 6 - Pathway Associations and GSEA p-values for cancer modules in ovarian cancer.

Module Genes	Significant Pathways	P-value	Number of Pathways
RB1, ABL1, TGFB1, TP53, NF1	Pathways<br>MAPK	3.43×10^{-3}<br>2.05×10^{-2}	2

IL6, STAT3, TNF, AKT1	FoxO	7.64×10^{-4}	2
	Pathways in cancer	4.88×10^{-2}	
BRAT1, PPARD, IGF1, AKT1	Pathways in cancer	1.18×10^{-2}	3
	FoxO	3.47×10^{-2}	
	mTOR	4.86×10^{-2}	
MTM1, MLST8, PTEN, MTOR	mTOR	1.64×10^{-4}	2
	PI3K-Akt	1.52×10^{-2}	
ERBB3, INS, PDGFRA, PDGFRB	MAPK	8.34×10^{-5}	3
	PI3K-Akt	1.64×10^{-4}	
	Ras	4.41×10^{-3}	
PIWIL2, ROCK2, MTOR, AKT1	Pathways in cancer	1.18×10^{-2}	3
	ErbB	1.42×10^{-2}	
	mTOR	4.86×10^{-2}	
MAP2K1, MAPK14, IGF1R, PIK3CA, IGF1, AKT1	FoxO	5.92×10^{-10}	9
	mTOR	5.53×10^{-7}	
	VEGF	1.53×10^{-6}	
	Ras	4.49×10^{-6}	
	MAPK	1.55×10^{-5}	
	PI3K-Akt	3.59×10^{-5}	
	Pathways in cancer	2.53×10^{-4}	
	ErbB	8.99×10^{-4}	
	Estrogen	3.81×10^{-3}	
FGFR4, JAK3, KDR, KIT	PI3K-Akt	1.64×10^{-4}	4
	Ras	4.41×10^{-3}	
	MAPK	9.25×10^{-3}	
	Pathways in cancer	4.88×10^{-2}	

ALK, EPHB3, EPHA2, EPHA1, TEK, KDR	Ras<br>MAPK	1.93×10^{-2}<br>4.00×10^{-2}	2
VAC14, ROR2, PDGFRB, CSF1R, ERBB3	MAPK<br>PI3K-Akt	2.05×10^{-2}<br>3.37×10^{-2}	2
RHOQ, MAPK3, PIK3R1, MAPK1	VEGF<br>ErbB<br>FoxO<br>Estrogen<br>mTOR<br>Ras<br>PI3K-Akt<br>Pathways in cancer	6.83×10^{-5}<br>2.00×10^{-4}<br>7.64×10^{-4}<br>8.56×10^{-4}<br>1.26×10^{-3}<br>4.41×10^{-3}<br>1.52×10^{-2}<br>4.88×10^{-2}	8
GHSR, EIF2AK3, MAPK1, MAPK3	VEGF<br>ErbB	1.51×10^{-2}<br>3.08×10^{-2}	2
PITPNM2, CSF1R, FLT4, KDR	Ras<br>MAPK<br>PI3K-Akt	1.03×10^{-3}<br>2.19×10^{-3}<br>3.62×10^{-3}	3
MAP2K2, SRF, CITED2, MAPK1, CTNNB1	MAPK<br>VEGF<br>ErbB	2.05×10^{-2}<br>2.29×10^{-2}<br>4.65×10^{-2}	3
ANGPTL4, KDR, FGFR1, IGF1R	Ras<br>MAPK<br>PI3K-Akt	4.41×10^{-3}<br>9.25×10^{-3}<br>1.52×10^{-2}	3
IFNL3, STAT3, NODAL, ACVR1, CITED2, HES1, SMAD3, SMAD4	FoxO<br>Pathways in cancer	1.06×10^{-2}<br>4.70×10^{-2}	2
PSMC3IP, RAD50, FLT1, MET, TREM2, EGFR, PDGFB	Ras<br>MAPK<br>PI3K-Akt	4.04×10^{-4}<br>1.08×10^{-3}<br>2.09×10^{-3}	3

EMP3, BNIP3, CASP2, FADD, CASP9, BAX, TP53	p53<br>Pathways in cancer	1.06×10^{-2}<br>4.70×10^{-2}	2
NFASC, SLC8A2, MAPK1, PDPK1, SNCA, MAPK3, AKT1	FoxO<br>mTOR<br>VEGF<br>ErbB<br>PI3K-Akt<br>Estrogen<br>Ras	8.90×10^{-5}<br>1.75×10^{-4}<br>5.32×10^{-4}<br>1.54×10^{-3}<br>4.68×10^{-3}<br>6.53×10^{-3}<br>3.28×10^{-2}	7

Table 7 - Pathway Associations and GSEA p-values for cancer modules in Cervis cancer.

Module Genes	Significant Pathways	P-value	Number of Pathways
PDGFRA, TP53, TGFB1, ABL1, CTNNB1, NF1, TNF	MAPK<br>Pathways in cancer<br>Ras	5.23×10^{-5}<br>8.34×10^{-4}<br>3.28×10^{-2}	3
SLC46A2, BRAF, MAP2K1, RAF1	ErbB<br>FoxO<br>mTOR<br>MAPK<br>VEGF<br>Pathways in cancer<br>Estrogen	4.63×10^{-5}<br>1.78×10^{-4}<br>2.96×10^{-4}<br>2.19×10^{-3}<br>7.03×10^{-3}<br>1.18×10^{-2}<br>3.74×10^{-2}	7
PKNOX1, CD4, KDR, NTRK1, ERBB4	MAPK<br>PI3K-Akt	9.25×10^{-3}<br>1.52×10^{-2}	2
GRIN2B, FYN, ERBB4, ERBB2, EGFR	ErbB<br>MAPK<br>PI3K-Akt	4.53×10^{-4}<br>2.05×10^{-2}<br>3.37×10^{-2}	3

TESC, WNT10B, WNT1, WNT4, WNT5A	mTOR<br>Wnt<br>Pathways in cancer	5.75×10^{-6}<br>9.18×10^{-6}<br>7.91×10^{-4}	3
ENPP3, PRKCD, MAP2K1, CDKN2A, TP53, CDKN1A	p53<br>Pathways in cancer	6.13×10^{-4}<br>9.78×10^{-3}	2
LRP1, EPHA3, RET, IGF1R, NTRK1, ERBB3	MAPK<br>PI3K-Akt	1.08×10^{-3}<br>2.09×10^{-3}	2
MLST8, RORA, TREM2, RPTOR, BCL2, MTOR	PI3K-Akt<br>mTOR	2.09×10^{-3}<br>5.63×10^{-3}	2
CNTN5, EPHA7, ATF2, PML, EPHA2, TP53	MAPK<br>PI3K-Akt	2.05×10^{-3}<br>3.37×10^{-2}	2
SMC5, NUP62, CDKN1A, CDKN1B	ErbB<br>FoxO	1.42×10^{-2}<br>3.47×10^{-2}	2
BTK, RELA, FGFR3, NBN, EPHB2, NTRK1	Ras<br>MAPK	1.93×10^{-2}<br>3.47×10^{-2}	2
PLK1, OGT, CDK1, HSP90AB1, HSP90AA1, PTGS2, AKT1, TGFB1	Pathways in cancer<br>FoxO<br>Estrogen	2.42×10^{-3}<br>1.06×10^{-2}<br>1.18×10^{-2}	3
HLA-DRB1, IL12B, PRKCA, RYK, ERBB4, TEK, FGFR3, EPHA1, RET	MAPK<br>PI3K-Akt	3.29×10^{-4}<br>7.50×10^{-4}	2
DYRK3, TTBK1, EGF, PTPRC, FGFR3, RELN, FLT4, KIT	PI3K-Akt<br>Ras<br>MAPK<br>Pathways in cancer	3.59×10^{-5}<br>4.04×10^{-4}<br>1.08×10^{-3}<br>9.78×10^{-3}	4
DBN1, TAOK1, CD36, HRAS, CCL5, FGFR1, KIT, PDGFB, PTPRC	MAPK<br>Ras<br>PI3K-Akt	1.56×10^{-4}<br>2.05×10^{-3}<br>1.04×10^{-2}	4

	Pathways in cancer	9.78×10^{-3}	
NRARP, IL2, LEP, PTGS2, AKT1, MAPK14, PIK3CA, XBP1	VEGF<br><br>FoxO<br><br>Pathways in cancer	8.08×10^{-6}<br><br>1.06×10^{-2}<br><br>4.70×10^{-2}	3

Table 8 - Pathway Associations and GSEA p-values for cancer modules in Adrenal cancer.

Module Genes	Significant Pathways	P-value	Number of Pathways
FGF10, MTOR, TP53, ABL1, NF1	Pathways in cancer<br><br>Ras<br><br>MAPK<br><br>PI3K-Akt<br><br>ErbB	3.43×10^{-3}<br><br>9.87×10^{-3}<br><br>2.05×10^{-2}<br><br>3.37×10^{-2}<br><br>4.65×10^{-2}	5
PRKCQ, RPTOR, XBP1, PIK3CA, MTOR	mTOR<br><br>PI3K-Akt<br><br>ErbB	2.85×10^{-3}<br><br>3.37×10^{-2}<br><br>4.65×10^{-2}	3
ZMIZ1, STAT3, PML, CDKN1A	Pathways in cancer<br><br>FoxO	1.18×10^{-5}<br><br>3.47×10^{-3}	2
CCR5, BANK1, DUSP6, MAPK3, PTPRC, ZFP36L1, MYC, BRAF, ABL1	ErbB<br><br>MAPK	5.86×10^{-5}<br><br>9.18×10^{-3}	2
PBX1, RAF1, MAPK3, FGF10	Ras<br><br>MAPK<br><br>VEGF<br><br>PI3K-Akt<br><br>ErbB<br><br>Pathways in cancer	4.41×10^{-3}<br><br>9.25×10^{-3}<br><br>1.51×10^{-2}<br><br>1.52×10^{-2}<br><br>3.08×10^{-2}<br><br>4.88×10^{-2}	6
AKT2, MAPK14, XBP1, MEF2C	MAPK<br><br>VEGF	9.25×10^{-3}<br><br>1.51×10^{-2}	2
MYO16, PIK3CB, PIK3CA, PTEN	FoxO	7.64×10^{-4}	6

	mTOR<br>VEGF<br>PI3K-Akt<br>ErbB<br>Pathways in cancer	2.36×10^{-3}<br>1.51×10^{-2}<br>1.52×10^{-2}<br>3.08×10^{-2}<br>4.88×10^{-2}	
CSNK2B, LMTK2, EPHA5, IGF1R, EPHB1, NTRK2, FGFR4, INSR, EPHA1	Ras<br>MAPK<br>PI3K-Akt	9.13×10^{-4}<br>2.42×10^{-3}<br>4.68×10^{-3}	3
SOCS7, PIK3C2B, IRS1, PIK3CG, AKT1, XBP1, PIK3CA	PI3K-Akt<br>FoxO<br>mTOR<br>VEGF	4.68×10^{-3}<br>5.84×10^{-3}<br>9.63×10^{-3}<br>4.73×10^{-2}	4

Table 9 - Pathway Associations and GSEA p-values for cancer modules in Kidney cancer.

Module Genes	Significant Pathways	P-value	Number of Pathways
PLD1, CDKN2A, CDKN1A, TP53	p53<br>Pathways in cancer	1.36×10^{-4}<br>7.91×10^{-4}	2
PIK3CB, IGF1R, AKT1, IGF1	FoxO<br>mTOR<br>Ras<br>PI3K-Akt<br>Pathways in cancer<br>MAPK<br>VEGF<br>ErbB	2.91×10^{-6}<br>5.75×10^{-6}<br>3.08×10^{-5}<br>1.64×10^{-4}<br>7.91×10^{-4}<br>9.25×10^{-3}<br>1.51×10^{-2}<br>3.08×10^{-2}	8
sPDX1, JUN, HES1, CITED2, TGFB1, SMAD4, TGFB2	Pathways in cancer<br>FoxO	8.34×10^{-4}<br>5.84×10^{-3}	2

FKBP10, SMAD2, FGF1, TGFB2, YAP1, TGFBR1	Pathways in cancer<br>MAPK	3.43×10^{-3}<br>2.05×10^{-2}	2
CAPRIN2, ANGPT1, PDGFB, VEGFA	Ras<br>MAPK<br>PI3K-Akt	1.03×10^{-3}<br>2.19×10^{-3}<br>3.37×10^{-2}	3
SEMA4D, HGF, ANGPT1, AGT, VEGFA	Ras<br>MAPK<br>PI3K-Akt	9.87×10^{-3}<br>2.05×10^{-2}<br>3.37×10^{-2}	3
SOX18, ZC3H12A, VEGFA, MAPK14, ANGPT1, LEP	MAPK<br>VEGF	9.25×10^{-3}<br>1.51×10^{-2}	2
PPM1F, NBN, DRD4, KDR, NTRK1, VEGFA	Ras<br>MAPK<br>VEGF<br>PI3K-Akt	9.87×10^{-3}<br>2.05×10^{-2}<br>2.29×10^{-2}<br>3.37×10^{-2}	4
FGFR1OP2, PPL, TGFBR2, SMAD3, SMAD2, TGFB2	FoxO<br>Pathways in cancer	7.64×10^{-4}<br>7.91×10^{-4}	2
GPR89A, ZAP70, ERBB4, FLT4, CSF1R, FGFR2	Ras<br>MAPK<br>PI3K-Akt	1.38×10^{-4}<br>3.70×10^{-4}<br>7.23×10^{-4}	3
WASF2, MMP2, VEGFA, PIK3CA, ANGPT1	Ras<br>VEGF<br>PI3K-Akt	9.87×10^{-3}<br>2.29×10^{-2}<br>3.37×10^{-2}	3
NOCT, MAPK10, MTOR, EZH2, PPARG	ErbB<br>Pathways in cancer	3.08×10^{-2}<br>4.48×10^{-2}	2
TNC, PDGFB, JAK2, PTK2	PI3K-Akt<br>Pathways in cancer	1.64×10^{-4}<br>4.88×10^{-2}	2
LONP1, EIF2S1, PIK3R1, PIK3R2, NF1	Ras<br>VEGF<br>ErbB	4.41×10^{-3}<br>1.51×10^{-2}<br>3.08×10^{-2}	3

S100A12, FLT1, FGF18, INSR, PDGFA, EPHA1, NTRK1	Ras<br>MAPK<br>PI3K-Akt	4.49×10^{-6}<br>1.55×10^{-5}<br>3.59×10^{-5}	3
COL2A1, PLEKHA1, PIK3R1, XBP1, IGF1, PIK3CA	PI3K-Akt<br>FoxO<br>mTOR<br>Ras<br>VEGF<br>ErbB	7.23×10^{-4}<br>1.72×10^{-3}<br>2.85×10^{-3}<br>9.87×10^{-3}<br>2.29×10^{-2}<br>4.65×10^{-2}	6
ARHGEF2, VEGFB, PAK2, PTK2B, EGF, ERBB4, SYK, PTPRC, FGFR2	MAPK<br>PI3K-Akt<br>Ras<br>ErbB	3.29×10^{-4}<br>7.50×10^{-4}<br>3.50×10^{-3}<br>4.07×10^{-3}	4
CCN1, EPHA7, CSF1R, TYRO3, EPHA1, FGFR3, EPHA2	Ras<br>MAPK	1.93×10^{-2}<br>4.00×10^{-2}	2
PYGO2, TDGF1, ANGPT1, VEGFA	Ras<br>MAPK<br>PI3K-Akt	2.02×10^{-2}<br>3.31×10^{-2}<br>4.88×10^{-2}	3
PPP1R10, PIK3R1, PPP3CA, SMO, CDKN1A	VEGF<br>ErbB<br>Pathways in cancer	1.51×10^{-2}<br>3.08×10^{-2}<br>4.88×10^{-2}	3
RGN, CDKN2A, CDKN1A, PML, HRAS	Pathways in cancer<br>p53<br>ErbB	3.43×10^{-3}<br>3.61×10^{-2}<br>4.65×10^{-2}	3

Table 10 - Pathway Associations and GSEA p-values for cancer modules in Corpus uteri cancer.

Module Genes	Significant Pathways	P-value	Number of Pathways
ATM, NF1, FGF10, TP53	MAPK<br>p53	9.25×10^{-3}<br>2.39×10^{-2}	2
JUN, CRK, CITED2, AKT1, GATA3	ErbB<br>MAPK	4.53×10^{-4}<br>2.05×10^{-2}	2
LGI1, RPTOR, AKT1, MTOR	mTOR<br>PI3K-Akt<br>ErbB	2.96×10^{-3}<br>3.62×10^{-3}<br>1.42×10^{-2}	3
LRRK2, ABL1, MTOR, TP53, AKT1	ErbB<br>Pathways in cancer<br>PI3K-Akt	4.53×10^{-4}<br>3.43×10^{-3}<br>3.37×10^{-2}	3
CDC25C, ISL1, CYP1A1, SMAD4, IGF1, GATA3, STAT3, AKT1	FoxO<br>Pathways in cancer	2.02×10^{-4}<br>4.70×10^{-2}	2
TPTE, PIK3R1, PIK3CA, AKT1	VEGF<br>ErbB<br>FoxO<br>Estrogen<br>mTOR<br>Ras<br>PI3K-Akt<br>Pathways in cancer	1.58×10^{-5}<br>4.63×10^{-5}<br>1.78×10^{-4}<br>2.00×10^{-4}<br>2.96×10^{-4}<br>1.03×10^{-3}<br>3.62×10^{-3}<br>1.18×10^{-2}	8
MAGI2, EPHA2, EPHA4, ERBB3, FLT3, EPHB2	PI3K-Akt<br>MAPK	2.09×10^{-3}<br>4.00×10^{-2}	2
NGEF, RASIP1, MLST8, RPTOR, BCL2, MTOR	PI3K-Akt<br>mTOR	2.09×10^{-3}<br>5.63×10^{-3}	2
HIPK3, LMTK2, NTRK1, FLT4, NTRK2, ERBB3	MAPK<br>PI3K-Akt<br>Ras	3.70×10^{-4}<br>7.23×10^{-4}	3

		9.87×10^{-4}	
DGKG, ALK, MET, NTRK1, FGFR1	Pathways in cancer<br><br>Ras<br><br>MAPK<br><br>PI3K-Akt	3.43×10^{-3}<br><br>9.87×10^{-3}<br><br>2.05×10^{-2}<br><br>3.37×10^{-2}	4
SRPK2, NR2E1, KDR, PTK2, FLT3, NOD2	VEGF<br><br>PI3K-Akt	1.51×10^{-2}<br><br>1.52×10^{-2}	2
BRD7, ZMIZ1, CDKN1A, CDKN1B	ErbB<br><br>FoxO	1.42×10^{-2}<br><br>3.47×10^{-2}	2
WNT8A, WNT6, WNT7A, CTNNB1	Wnt<br><br>Pathways in cancer<br><br>mTOR	9.18×10^{-6}<br><br>7.91×10^{-4}<br><br>1.26×10^{-3}	3
RAPGEF3, PIK3CA, IL6, PTGS2	VEGF<br><br>Pathways in cancer	1.51×10^{-2}<br><br>4.88×10^{-2}	2
ARTN, CTNNA1, BCL2L11, GSK3A, ATM, ATF2, TGFB2, CASP3, CTNNB1	Pathways in cancer<br><br>MAPK<br><br>FoxO	5.01×10^{-3}<br><br>9.18×10^{-3}<br><br>1.52×10^{-2}	3

Table 11 - Pathway Associations and GSEA p-values for cancer modules in Prostate cancer.

Module Genes	Significant Pathways	P-value	Number of Pathways
WNT5A, MTOR, NF1, ABL1, TP53	Pathways in cancer<br><br>ErbB	3.43×10^{-3}<br><br>4.65×10^{-2}	2
PIK3R1, MAPK14, GLI3, NF1, TP53	MAPK<br><br>VEGF	2.05×10^{-2}<br><br>2.29×10^{-2}	2
SEMA5A, WNT3, WNT5A, WNT3A	mTOR<br><br>Wnt<br><br>Pathways	1.26×10^{-3}<br><br>1.79×10^{-3}<br><br>4.88×10^{-2}	3
S100A9, MLST8, RPTOR, MTOR	mTOR<br><br>PI3K-Akt	1.26×10^{-3}<br><br>1.52×10^{-2}	2

ZNF580, FGF1, WNT5A, WNT3A	Pathways in cancer<br>mTOR	1.18×10^{-2}<br>4.86×10^{-2}	2
INPP5K, EPHA2, PIK3R1, AKT1, ERBB2	ErbB<br>PI3K-Akt<br>Ras<br>MAPK<br>VEGF	4.53×10^{-4}<br>7.23×10^{-4}<br>9.87×10^{-3}<br>2.05×10^{-2}<br>2.29×10^{-2}	5
DSP, CDKN1A, CASP3, CDKN2A	p53<br>Pathways in cancer	1.36×10^{-4}<br>4.88×10^{-2}	2
ACER2, CDKN2A, PRKN, CDKN1A, CDKN1B	p53<br>ErbB	3.61×10^{-2}<br>4.65×10^{-2}	2
PLCB3, PIRT, CSF1R, TEK, FLT1, EPHA3	Ras<br>MAPK<br>PI3K-Akt	9.87×10^{-4}<br>2.05×10^{-2}<br>3.37×10^{-2}	3
SETD9, SGK1, NOP53, MTOR, AKT1	mTOR<br>PI3K-Akt<br>ErbB<br>FoxO	2.96×10^{-4}<br>3.62×10^{-3}<br>1.42×10^{-2}<br>3.47×10^{-2}	4
FAS, BAD, BMP7, PTGS2	VEGF<br>Pathways in cancer	1.51×10^{-2}<br>4.88×10^{-2}	2
SPRY4, BTN2A2, EDN1, IGF1, PTEN	p53<br>Pathways in cancer<br>FoxO<br>mTOR	1.10×10^{-2}<br>1.18×10^{-2}<br>3.47×10^{-2}<br>4.86×10^{-2}	4
ITGAV, NRG1, FGF1, AQP1, TGFB3, TGFBR2, FGFR2, ITGB3	Pathways in cancer<br>MAPK<br>PI3K-Akt	2.42×10^{-3}<br>5.42×10^{-3}<br>1.04×10^{-2}	3

Table 12 - Pathway Associations and GSEA p-values for cancer modules in Brain cancer.

Module Genes	Significant Pathways	P-value	Number of Pathways
SMAD4, TGFB1, CTNNB1, NF1, TP53	Pathways in cancer<br>Wnt<br>MAPK	3.43×10^{-3}<br>4.03×10^{-3}<br>2.05×10^{-2}	3
SRC, TNF, BCL2, TP53, NF1	MAPK<br>p53	2.05×10^{-3}<br>3.61×10^{-2}	2
CSF1R, PTK2, TGFB2, PDGFRA, SRC	Pathways in cancer<br>MAPK<br>VEGF<br>PI3K-Akt<br>ErbB	3.43×10^{-3}<br>2.05×10^{-2}<br>2.29×10^{-2}<br>3.37×10^{-2}<br>4.65×10^{-2}	5
PLEC, PPARD, TGFB2, IGF1, SMAD4	FoxO<br>Pathways in cancer	7.64×10^{-4}<br>7.91×10^{-4}	2
CAT, FLT3, PTK2, FGFR1, NTRK1	PI3K-Akt<br>Pathways in cancer<br>Ras<br>MAPK	7.23×10^{-4}<br>3.43×10^{-3}<br>9.87×10^{-3}<br>2.05×10^{-2}	4
NACA2, NACA, FGFR2, NOG, TGFBR1, TGFBR2, PDGFRB, SMAD4	Pathways in cancer<br>MAPK<br>FoxO	8.34×10^{-4}<br>2.42×10^{-3}<br>5.84×10^{-3}	3
ROMO1, WRN, ATM, CDKN1A	p53<br>FoxO	1.98×10^{-3}<br>6.26×10^{-3}	2
NUP188, PML, CDKN1A, CDKN2A	p53<br>Pathways in cancer	2.39×10^{-2}<br>4.88×10^{-2}	2
MAP2K2, SETX, IL1B, RAF1,	MAPK<br>FoxO	1.56×10^{-4}<br>1.06×10^{-2}	3

TGFB1, PTPRC, FGFR1, PTK2B	Pathways in cancer	4.70×10^{-2}	
IL12RB2, DYRK1A, ABL1, PTK2, PDGFB	Pathways in cancer<br>ErbB signaling pathway	7.91×10^{-4}<br>3.08×10^{-2}	2
AGRP, MAPK14, PIK3R1, AKT1, IGF1	FoxO<br>VEGF<br>mTOR<br>Ras<br>MAPK<br>PI3K-Akt<br>ErbB	1.3×10^{-5}<br>1.55×10^{-4}<br>2.85×10^{-3}<br>9.87×10^{-3}<br>2.05×10^{-2}<br>3.37×10^{-2}<br>4.65×10^{-2}	7
EPGN, BRAF, MEF2C, HRAS, IL1B	MAPK<br>ErbB	8.30×10^{-4}<br>3.08×10^{-2}	2
PROK1, PDGFD, VEGFA, NRP1, ANGPT1	Ras<br>MAPK<br>PI3K-Akt	4.41×10^{-3}<br>9.25×10^{-3}<br>1.52×10^{-2}	3

Table 13 - Pathway Associations and GSEA p-values for cancer modules in Liver cancer.

Module Genes	Significant Pathways	P-value	Number of Pathways
RB1, ABL1, TP53, SRC, TGFB1	Pathways in cancer<br>ErbB	3.43×10^{-3}<br>4.65×10^{-2}	2
IL6, P2RX7, TGFB1, SMAD3	FoxO<br>Pathways in cancer	7.64×10^{-4}<br>4.88×10^{-2}	2
PRDX2, MAP2K1, RAF1, BRAF	ErbB<br>FoxO<br>mTOR	4.60×10^{-5}<br>1.78×10^{-4}<br>2.96×10^{-4}	7

	MAPK<br>VEGF<br>Pathways in cancer<br>Estrogen	2.19×10^{-3}<br>7.03×10^{-3}<br>1.18×10^{-2}<br>3.74×10^{-2}	
CHEK2, TREX1, E2F1, TP53, BCL2	p53<br>Pathways in cancer	1.36×10^{-4}<br>4.88×10^{-2}	2
FABP4, IGF1R, PIK3R1, AKT1	FoxO<br>mTOR<br>Ras<br>VEGF<br>PI3K-Akt<br>ErbB<br>Pathways in cance	7.64×10^{-4}<br>1.26×10^{-3}<br>4.41×10^{-3}<br>1.51×10^{-2}<br>1.52×10^{-2}<br>3.08×10^{-2}<br>4.88×10^{-2}	7
F2RL1, PTK2, NTRK1, JAK2	PI3K-Akt<br>Pathways in cancer	1.52×10^{-2}<br>4.88×10^{-2}	2
PCDH12, MAP2K1, EDN1, MAPK3, BRAF, MAPK1	ErbB<br>FoxO<br>mTOR<br>Pathways in cancer<br>VEGF<br>MAPK<br>Estrogen<br>Ras<br>PI3K-Akt	2.00×10^{-6}<br>1.30×10^{-5}<br>2.60×10^{-5}<br>4.50×10^{-5}<br>1.55×10^{-4}<br>3.70×10^{-4}<br>1.92×10^{-3}<br>9.87×10^{-3}<br>3.37×10^{-2}	9
DRD5, RAF1, MAP2K1, MAP3K5	MAPK<br>VEGF	9.25×10^{-3}<br>1.51×10^{-2}	3

	ErbB	3.08×10^{-2}	
TGFA, PDGFRA, NOG, EGFR, FGFR1	Ras<br>signaling pathway<br>MAPK PI3K-Akt<br>Pathways in cancer<br>ErbB	1.38×10^{-4}<br>3.70×10^{-4}<br>7.23×10^{-4}<br>3.43×10^{-3}<br>4.65×10^{-2}	5
PRR5L, ERBB4, TEK, PDGFB	MAPK<br>PI3K-Akt	9.25×10^{-3}<br>1.52×10^{-2}	2
RHOQ, PIK3R3, IRS1, INSR, INS, AKT1	FoxO<br>mTOR<br>PI3K-Akt<br>Ras<br>VEGF<br>MAPK	2.00×10^{-6}<br>6.00×10^{-6}<br>3.60×10^{-5}<br>4.04×10^{-4}<br>3.43×10^{-2}<br>4.00×10^{-2}	6
STXBP4, PIK3R2, PDPK1, GPLD1, AKT1	FoxO<br>mTOR<br>VEGF<br>PI3K-Akt<br>ErbB	7.64×10^{-4}<br>1.26×10^{-3}<br>1.51×10^{-2}<br>1.52×10^{-2}<br>3.08×10^{-2}	5
WNT2, DDR2, EPHB2, PDGFRA, NTRK1, PDGFB	Pathways in cancer<br>Ras<br>MAPK<br>PI3K-Akt	3.43×10^{-3}<br>9.87×10^{-3}<br>2.05×10^{-2}<br>3.37×10^{-2}	4
TNIK, SMAD1, MYC, MAPK3, MAP2K1	ErbB<br>MAPK<br>VEGF	2.00×10^{-4}<br>9.25×10^{-3}<br>1.51×10^{-2}	5

	PI3K-Akt<br>Pathways in cancer	1.52×10^{-2}<br>4.88×10^{-2}	
GCKR, IPO5, CDKN1A, CDKN1B	ErbB<br>FoxO	1.42×10^{-2}<br>3.47×10^{-2}	2
DUSP6, HSF1, SETX, MAP2K1, MAPK3	MAPK<br>VEGF<br>ErbB	2.05×10^{-2}<br>2.29×10^{-2}<br>4.65×10^{-2}	3
CLCA1, DNMT3A, BAD, CASP3, HIPK2, CDKN1B, CDKN1A	ErbB<br>Pathways in cancer	1.54×10^{-3}<br>2.15×10^{-2}	2
WWTR1, EPHA1, NTRK2, ERBB4, KIT, ERBB2	MAPK<br>PI3K-Akt	1.08×10^{-3}<br>2.09×10^{-3}	2
EPHA10, ZNF16, FGFR2, IGF1R	Ras<br>MAPK<br>PI3K-Akt	2.02×10^{-2}<br>3.31×10^{-2}<br>4.64×10^{-2}	3
LSS, FBXO7, CDKN2A, NF2, CDKN1A, HRAS	p53<br>ErbB	3.61×10^{-2}<br>4.64×10^{-2}	2
LCMT1, OPRD1, CDKN1B, APC, PTEN, CDKN1A	FoxO<br>Pathways in cancer<br>PI3K-Akt<br>p53<br>ErbB	1.72×10^{-3}<br>3.43×10^{-3}<br>3.37×10^{-2}<br>3.61×10^{-2}<br>4.65×10^{-2}	5
CCNI2, CDK5RAP3, CDKN2B, DAB2IP, CDKN2A, CDKN1A	p53<br>Pathways in cancer	2.39×10^{-2}<br>4.88×10^{-2}	2
RPS14, GATA1, HCLS1, NTRK3, INSR, ERBB4, ANGPT1, JAK2, TEK	PI3K-Akt<br>MAPK	3.57×10^{-4}<br>5.42×10^{-3}	2

PLCD4, PLCL1, CSF1R, INSR, PTK2B, FGFR1, IGF1R, KIT	Ras<br>MAPK<br>PI3K-Akt<br>Pathway in cancer	4.56×10^{-5}<br>1.55×10^{-4}<br>3.56×10^{-4}<br>4.70×10^{-2}	4
PRDX1, MAP2K1, PIK3CA, RAF1, CAV1	VEGF<br>ErbB<br>Fox0<br>Estrogen<br>mTOR<br>Ras<br>PI3K-Akt	1.55×10^{-4}<br>4.52×10^{-4}<br>1.72×10^{-3}<br>1.92×10^{-3}<br>2.85×10^{-3}<br>9.87×10^{-3}<br>3.37×10^{-2}	7
KDM5C, DRD4, CSF1R, PTPRC, FYN, FGFR1, NTRK1, EPHB2, ERBB2	MAPK<br>PI3K-Akt<br>Pathway in cancer	5.42×10^{-3}<br>1.04×10^{-2}<br>4.70×10^{-2}	3
NFASC, SEMA3E, HGF, FLT3, PDGFC, NTRK3, IGF1R, NTRK2, ERBB4, KDR	MAPK<br>PI3K-Akt<br>Ras	4.43×10^{-7}<br>1.42×10^{-6}<br>4.97×10^{-6}	3
LMTK2, TTBK1, NBN, EPHA4, NTRK1, KDR, NTRK2	Ras<br>MAPK<br>PI3K-Akt	9.87×10^{-3}<br>2.05×10^{-2}<br>3.37×10^{-2}	3

Table 14 - Pathway Associations and GSEA p-values for cancer modules in Thyroid cancer.

**Module Genes**	**Significant Pathways**	**P-value**	**Number of Pathways**

IGF1, ABL1, NF1, WNT5A, CTNNB1, TGFB1, TP53	Pathways in cancer<br>MAPK<br>Wnt<br>Ras	1.80×10^{-5}<br>2.42×10^{-3}<br>1.35×10^{-2}<br>3.28×10^{-2}	4
IL6, TGFB3, BMP7, TGFBR1	FoxO<br>Pathways	7.64×10^{-4}<br>4.88×10^{-2}	2
KAT5, LIG4, HRAS, CDKN1A, TP53	PI3K-Akt<br>p53<br>ErbB	3.37×10^{-2}<br>3.61×10^{-2}<br>4.65×10^{-2}	3
F2RL1, PDGFB, FLT3, JAK2	PI3K-Akt<br>Pathways in cancer	1.52×10^{-2}<br>4.88×10^{-2}	2
ARHGAP22, PIK3CA, MAPK14, XBP1, AKT1	VEGF<br>FoxO<br>ErbB	6.80×10^{-5}<br>7.64×10^{-4}<br>3.08×10^{-2}	3
CHGA, PIK3CG, PIK3CA, IGF1, PTEN	PI3K-Akt<br>FoxO<br>mTOR<br>p53<br>Pathways in cancer	1.64×10^{-4}<br>7.64×10^{-4}<br>1.26×10^{-3}<br>2.39×10^{-2}<br>4.88×10^{-2}	5
LRSAM1, CBL, NKX3-1, IGF1, INS, PPARD, ERBB3	Pathways in cancer<br>MAPK	9.78×10^{-3}<br>4.00×10^{-2}	2
PTPN22, PIK3CD, AKT1, ZFP36L1	VEGF<br>ErbB<br>FoxO<br>Estrogen<br>mTOR	7.03×10^{-3}<br>1.42×10^{-2}<br>3.47×10^{-2}<br>3.74×10^{-2}<br>4.86×10^{-2}	5
SOS2, PLD1, RAPGEF2, KRAS, NF1	Ras<br>MAPK	1.38×10^{-4}<br>3.70×10^{-4}	3

	ErbB	4.65×10^{-2}	
ITGA7, PTPN11, PTPN6, PTK2, TEK, FGFR1	PI3K-Akt<br>Ras	2.09×10^{-3}<br>1.93×10^{-2}	2
NUP153, NFKBIA, TRAF3IP2, AKT1, PIK3R1	VEGF<br>ErbB	2.29×10^{-2}<br>4.65×10^{-2}	2
FAM20C, WNT10B, WNT1, WNT3A	mTOR<br>Wnt<br>Pathways in cancer	2.96×10^{-4}<br>4.20×10^{-4}<br>1.18×10^{-2}	3

Table 15 - Pathway Associations and GSEA p-values for cancer modules in Colon cancer.

**Module Genes**	**Significant Pathways**	**P-value**	**Number of Pathways**
HDAC6, CDKN2A, PML, CDKN1A, ABL1	Pathways in cancer<br>p53<br>ErbB	3.43×10^{-3}<br>3.61×10^{-2}<br>4.65×10^{-2}	3
LTBP4, HIPK2, PPARG, BCL2, TP53	p53<br>Pathways in cancer	2.39×10^{-2}<br>4.88×10^{-2}	2
FCHO1, BCL10, EPHB4, DDR1, FLT1, FGFR3, FGFR1, EPHA3, EPHB2, ERBB4	MAPK<br>PI3K-Akt	5.42×10^{-3}<br>1.04×10^{-2}	2
GRIK5, EPHB1, NTRK2, ERBB3, TYRO3, FGFR3, TEK, KIT, NTRK3	MAPK<br>PI3K-Akt<br>Ras	3.29×10^{-4}<br>7.50×10^{-4}<br>3.50×10^{-3}	3
PHPT1, CSF1R, FLT4, FGFR1	Ras<br>MAPK<br>PI3K-Akt<br>Pathways in cancer	1.03×10^{-3}<br>2.19×10^{-3}<br>3.62×10^{-3}<br>1.18×10^{-2}	4

GDF2, TGFB3, SMAD4, BMP7, TGFBR1, BMP2	FoxO<br><br>Pathways	3.41×10^{-3}<br><br>9.78×10^{-3}	2
CDC25A, WNT10B, WNT1, WNT5A, WNT3A	mTOR<br><br>Wnt<br><br>Pathways in cancer	2.60×10^{-5}<br><br>4.10×10^{-5}<br><br>3.43×10^{-3}	3
DCBLD2, PPARD, IGF1, SMAD4	Pathways in cancer<br><br>FoxO	1.18×10^{-2}<br><br>3.47×10^{-2}	2
WFDC1, NPM1, MAPK8, MAPK14, HRAS, TP53	MAPK<br><br>FoxO<br><br>VEGF<br><br>ErbB<br><br>Pathways in cancer	8.30×10^{-4}<br><br>7.64×10^{-4}<br><br>1.51×10^{-2}<br><br>3.08×10^{-2}<br><br>4.88×10^{-2}	5

Table 16 - Pathway Associations and GSEA p-values for cancer modules in Skin cancer.
